# A Nationwide Survey of Animal Science Students’ Perceptions of Animal Welfare across Different Animal Categories at Institutions in the United States

**DOI:** 10.3390/ani12172294

**Published:** 2022-09-05

**Authors:** Paxton Sullivan, Sage Mijares, Melissa Davis, Katrina Oselinsky, Catie Cramer, Noa Román-Muñiz, Lorann Stallones, Lily Edwards-Callaway

**Affiliations:** 1 Department of Animal Sciences, Colorado State University, Fort Collins, CO 80523-1171, USA; 2 Department of Psychology, Colorado State University, Fort Collins, CO 80523-1171, USA

**Keywords:** agricultural animals, companion animals, equids, horses, well-being, wildlife

## Abstract

**Simple Summary:**

The field of animal welfare science studies the well-being of animals, including an animal’s physical and emotional state. Previous studies have investigated veterinary students’ perceptions of animal welfare, although few studies have focused on animal science students’ perceptions of the topic. The aim of this study was to determine if animal science students’ perceptions of animal welfare differed across a multitude of animal categories, including agricultural animals, cats and dogs, horses and other equids, research animals, and wildlife. Results indicate that most survey respondents agreed that animal welfare was important for all animal categories. In qualitative analyses of the open-ended survey questions, basic needs and human interaction were identified by respondents as key welfare components for all animal categories; however, the frequency with which identified welfare themes was mentioned differed by category. Results also highlighted perception differences within different agricultural animal species; fewer respondents agreed that poultry and swine are raised with an appropriate level of welfare compared to dairy and beef cattle. Understanding animal science students’ perceptions of animal welfare may lead to a greater understanding of how they will assess and manage animal welfare issues as part of their future careers in the agricultural and animal-related sectors.

**Abstract:**

Animal welfare is an increasingly important topic across multiple academic disciplines; however, few studies have investigated student perceptions of animal welfare outside of veterinary medicine. The objective of the study was to evaluate animal science students’ perceptions of animal welfare to determine if perceptions differ across animal categories. An online survey was distributed to animal science programs at institutions across the United States. Quantitative and qualitative analyses were performed on 624 responses. Almost all respondents agreed welfare was important for all animal categories (≥97%). The survey asked respondents to rate the level of importance of 12 welfare parameters and there was evidence that the level of importance differed by animal category (*p* < 0.0001), e.g., fewer respondents indicated having positive interactions with humans was important for agricultural animals. In a subset of questions about agricultural animals, fewer respondents agreed that swine (325, 52.1%) and poultry (268, 43.0%) are raised with an appropriate level of welfare compared to dairy (425, 68.1%) and beef cattle (421, 67.5%). Four free-response questions asked respondents to report their general perceptions of welfare. Thematic analysis identified multiple themes, such as basic needs and human interaction, with most responses (75%) including two or more themes.

## 1. Introduction

Animal welfare is a topic that receives significant attention in the public domain and is increasingly regarded as a deciding factor for society about whether animal care and use practices are acceptable and, therefore, sustainable [1,2,3,4]. Public perceptions of animal welfare are continually evolving, influencing laws about animal usage [1] and impacting consumers’ purchasing decisions [2,3,4]. Furthermore, public concern and perceptions about animal welfare dictate the way animals are used and cared for in many capacities, for which several contemporary examples exist. SeaWorld announced it would halt its orca breeding program and focus on conservation efforts after mass public outcry following the release of the documentary *Blackfish* [5]. Additionally, in recent years, many states across the United States have passed legislation banning the use of heavily scrutinized management practices, such as confined housing systems for livestock and poultry [6] and the banning of activities related to horse slaughter [7]. This increased awareness and concern for animal welfare is not just isolated to the United States; in a 2016 survey of European citizens, over half of the citizens surveyed expressed a strong concern for animal welfare [8]. These societal concerns about animal welfare are found in much of the current literature, particularly outside of the US (Canada, [9]; Ireland, [4]; Mexico, [10]; South America [11]), and have driven new legislation regarding animal care and use practices, for example, the banning of caged layers in the European Union [12] and painful procedures in piglets in Norway and in Sweden [13]. Concomitantly, perceptions of animal welfare are complex and often influenced by a multitude of environmental and social factors [14,15,16,17]. While different stakeholders in animal industries increasingly value animal wellbeing, they tend to assess animal welfare differently; some individuals may value health and productivity as indicators of good welfare while others place a greater emphasis on emotional wellbeing and the ability for animals to engage in natural behaviors. As the field of animal welfare science continues to progress and evolve, so too has the way that industry stakeholders (e.g., veterinarians, livestock producers, consumers) conceptualize animal welfare, who often use multiple indicators of welfare to make more holistic welfare assessments.

Although all animal industries face unique animal welfare challenges, the welfare of agricultural animals is particularly relevant as consumer trends and company-specific standards of animal care are informed largely by the public’s increased awareness and interest in where their food comes from. Previous research has explored how perceptions of and attitudes towards animal welfare issues vary across animal species and industries. Heleski and Zanella [18] reported perception differences between varying species in a survey of undergraduate students; to illustrate, students perceived that horses could feel pain and experience boredom in ways similar to humans more than other agricultural species (i.e., poultry, cattle, sheep, and pigs). Additionally, a multitude of work in this space has discovered that a variety of stakeholder groups, including animal science students and faculty, veterinarians, and consumers, perceive welfare more positively and express greater comfort with less intensive production systems (e.g., beef and sheep systems) compared to more intensive systems (e.g., layers, poultry, and swine systems) [3,4,15,19]. Studies have also found that a positive, hands-on learning environment and competencies in animal welfare can strengthen empathy towards animals [20]. 

While the disparities between perceptions of animal welfare among individuals and differences in methodologies used for assessing welfare have implications for animal science students in higher education, animal welfare education is not a foundational component of most animal science degree programs. In a survey of graduate and undergraduate students in animal science programs in the United States, Mijares and others [21] reported the majority of students surveyed had never taken an animal welfare class, and of the top ten land-grant universities in the United States, only six offered animal welfare courses [18]. Providing formal and extra-curricular opportunities for animal science students to learn about animal welfare is important because these students will likely work in agricultural and animal-related sectors. Moreover, many will regularly assess and manage animal welfare issues as part of their careers, so exposure to animal welfare concepts during their education is crucial. Animal welfare-related courses and training have been recognized as an important component of veterinary medicine curricula by organizations like the American College of Animal Welfare (ACAW) [22] and the American Association of Veterinary Colleges (AAVMC) [23]; despite this, studies have suggested that animal welfare education is still lacking in veterinary schools [24,25]. Mota-Rojas et al. [26] review different approaches to teaching animal welfare specifically in Latin America, noting the importance of integrating diverse curricular approaches to strengthen animal welfare competencies of veterinary students. Although some studies exploring undergraduate students’ perceptions about animal welfare education and topics exist [27,28], the majority of research has focused on veterinary students [25,29,30,31,32]. Broader animal welfare education would help equip students with a working knowledge of animal welfare science in order to adequately evaluate a wide range of welfare challenges with the ultimate goal of helping to create solutions to improve animal welfare regardless of the animal industry.

The first iteration of this survey study [21] was written with a particular emphasis on agricultural animal welfare since agricultural animal production is a major component of the animal science curricula; however, this particular manuscript investigated perceptions of animal welfare across multiple animal categories and industries. The authors recognize that welfare is a critical component of animal care and use in all animal-related industries and thus, that concept is reflected in this manuscript. The objective of the current study was to evaluate attitudes and perceptions of animal welfare of students enrolled in animal science programs at institutions across the United States and determine if welfare considerations differ across animal categories (e.g., agricultural animals, cats and dogs, horses and other equids, research animals, and wildlife).

## 2. Materials and Methods

This study was approved by the Colorado State University (CSU) Institutional Review Board (#21-10558H) prior to project initiation. This paper presents data not previously reported from a larger survey described in Mijares et al. [21] for which the methodology is the same.

### 2.1. Study Population and Survey Development

This project was conducted in the spring of 2021. This survey was intended for undergraduate and graduate students at colleges and universities across the United States pursuing animal science degrees. The survey was developed in Qualtrics survey software (Qualtrics, Provo, UT, USA). The online survey information was distributed via a listserv composed of 84 email addresses of administrators affiliated with animal science departments. In the email, researchers requested that administrators share the survey with undergraduate and graduate students within their departments. The email included the direct link to the survey. The email also offered a USD 25 gift card to the first 100 respondents who completed the survey, which was emailed to an address provided that was not linked to survey responses. The only two forced answer questions in the survey were for obtaining consent to participate and confirming that respondents were enrolled in an animal science degree program.

The entire survey included 46 questions, but due to the implementation of branching logic, a variable number of questions were presented to each individual. The subset of questions presented here focused on perceptions of animal welfare across different animal categories. Five Likert scale questions asking respondents to indicate their agreement with animal welfare being an important consideration for different conditions (i.e., agricultural animal production, owning a cat or dog, owning a horse or other equid, when conducting research on animals, and for wildlife) were included. Possible responses for Likert scale questions included strongly agree, agree, neither agree or disagree, disagree, or strongly disagree. Respondents were asked to indicate the importance (i.e., extremely important, very important, moderately important, slightly important, not at all important, I don’t know) of a variety of parameters (e.g., room to move around freely, freedom from fear and distress) for three different animal categories (i.e., animals raised for food and fiber, a dog or cat, and horses or other equids). The parameters used were adapted from Heleski and Zanella [18]. One question, from Heleski and others [33], asking respondents to indicate their level of agreement with the statement “predominant methods presently used to raise each animal category below (i.e., dairy cattle, beef cattle, poultry, swine, sheep and goats) for food and/or fiber provide an appropriate level of animal welfare”, was included. Lastly, four free-response questions related to what animal welfare means generally and what different animal categories (i.e., agricultural animal, dog or cat, and horse or other equid) need to have a good life were included in the analysis. Demographic questions were included at the end of the survey. The entire survey is available in the Supplementary Materials.

### 2.2. Statistical Analysis

#### 2.2.1. Quantitative Analysis

Respondents were asked to state their level of agreement with the statement “animal welfare is an important consideration” for each of the following: agricultural animal production, owning a cat or dog, owning a horse or other equid, conducting research with animals, and for wildlife. Likert responses were collapsed into ‘agree’ (strongly agree + agree) vs. ‘did not agree’ (neither agree or disagree + disagree + strongly disagree). A logistic regression (PROC GLIMMIX in SAS, version 9.4) was fit to determine if the proportion of respondents who agreed that welfare was important differed between species. Respondent was included as a random effect to account for a lack of independence between responses.

Twelve factors related to animal welfare were listed and respondents were asked to indicate the level of importance (i.e., extremely important, very important, moderately important, slightly important, not at all important, I don’t know) for each factor for food and fiber animals, dogs and cats, and equids. A chi-square test was used to determine if there was a difference in respondents’ perceived level of importance for a given welfare factor among animal categories. Phi (*φ*) was selected as the measure of effect size, with phi values ≥ 0.5 a large effect.

For the statement “the way in which animals are currently raised provides an appropriate level of animal welfare,” respondents were asked to indicate the level of agreement for each of the following: dairy cattle, beef cattle, poultry, swine, and sheep and goats. Likert responses were collapsed into three categories: ‘agree’ (strongly agree + agree), ‘did not agree’ (neither agree or disagree + disagree + strongly disagree), and “not enough information to decide.” A logistic regression (PROC GLIMMIX in SAS) was fit to determine if the proportion of respondents who agreed, did not agree, or did not have enough information to decide differed between species; this model used respondent as a random effect to account for repeated measures.

#### 2.2.2. Qualitative Analysis

Thematic analysis was conducted on the four open-response survey questions. Analysis was conducted as described by Braun and Clarke [34]. Questions were analyzed in two sections due to the relatedness of three of the questions; one section included one question (“What does animal welfare mean to you?”) and the second section included three questions (“In your opinion, what does an [(1) agricultural animal (cattle, sheep, goat, pig, or poultry intended for food and fiber use), (2) dog or cat, and (3) horse or other equid] need in order to have a good life?”). All co-authors reviewed survey responses and developed initial themes as a group. A subset of individuals from this group created two codebooks, one for the individual question and one for the trio of questions. Survey responses were coded independently by three researchers and the same three coders analyzed all four questions. All three coders had varying levels of experience in animal health and welfare. One coder considers herself an animal welfare scientist having both experience working in the livestock industry and academia conducting teaching and research activities in the field of animal welfare for over a decade. The second coder is working towards her doctoral degree in Animal Science studying animal welfare and meat quality with a specific focus on preslaughter management of cattle. The third coder has a bachelor’s degree in Animal Science, is currently a fourth-year veterinary student, and has been involved in animal welfare research for the past five years.

To validate the coding, the initial total agreement of all codes needed to be 75% or greater before continuing. Generally, any two coders were in full agreement for each response with the third coder in partial agreement but having an additional theme present or one absent as there were a significant number of multi-themed responses. Additionally, within multi-themed responses, all three coders had the majority of themes identified (i.e., if a response had four themes present, all three coders would have three of the same identified and differed on one prior to discussion). Differences between coders were discussed and full agreement was reached for all survey responses from the four open-response survey questions. To express the frequency of themes present for each question, the number of times the theme was mentioned was divided by the total number of responses for that question.

## 3. Results

Analyses included 624 survey respondents. Calculating the actual response rate is not feasible due to the nature of how the survey was distributed, i.e., to administrators who subsequently shared the link with a greater list of individuals. The survey was shared at 24 unique higher education institutions across the United States. Detailed demographics are reported by Mijares and others [21]. In brief, the majority of respondents were undergraduate students (487, 78.0%) with 19.9% (124) enrolled in a graduate program. The majority of students were female (537, 86.1%) and between the ages of 18 and 24 (536, 85.9%). Slightly less than half the students were from a suburban background (287, 46.0%) and 42.3% (264) and 9.9% (62) were from rural and urban backgrounds, respectively. Additionally, as reported in Mijares et al. [21], nearly two-thirds (60.7%, 379) of respondents had not taken an animal welfare course.

### 3.1. Quantitative Results

The vast majority (≥97%) of respondents agreed that welfare was important for all animal categories (Table 1). Although fewer respondents (608, 97%) agreed that welfare was important for wildlife compared to all other animal categories (*p* < 0.005; Table 1) the percentage of those agreeing still represented the majority of respondents.

For each of the 12 factors related to welfare, there was evidence that the level of importance differed within animal categories (*p* < 0.0001; Table 2); effect sizes ranged from 0.6 to 1.6, indicating a large effect size. For all three animal categories, the majority of respondents indicated that all factors related to welfare were “extremely important,” except “ability for choice and control within their environment;” approximately half of respondents stated that the “ability for choice and control within their environment” was “extremely important” for dogs and cats and equids (48.5%, 301 and 48.6%, 297, respectively), whereas only 33.6% (207) of respondents stated this was extremely important for food and fiber animals (Table 2). Fewer respondents consistently indicated that a given welfare factor was “extremely important” for food and fiber compared to cats and dogs and equids, with the exception of “freedom from thirst,” whereby over 91% of respondents stated this was extremely important for all animal categories (Table 2). No one selected “I don’t know” when asked to indicate the level of importance of the factors related to welfare for any of the species. Interestingly, “having a life worth living” and “ability for choice and control within their environment” had the most respondents indicate that these were “not at all” important for all animal categories compared to other welfare factors (Table 2).

The level of agreement with the statement “the way in which animals are currently raised provides an appropriate level of animal welfare” was similar for dairy cattle and beef cattle (*p* > 0.1; Table 3). However, the fewest people agreed that poultry (43%, 268) were raised in a way that provides an appropriate level of animal welfare compared to dairy and beef cattle (68.1%, 425; 67.5%, 421; *p* < 0.01, respectively), followed by swine (52.1%, 325; Table 3). Interestingly, almost 18% (111) of respondents indicated that they did not have enough information to decide if the way in which sheep and goats are raised provides an appropriate level of welfare, which was more than twice as many respondents for all other animal categories (*p* < 0.01; Table 3).

### 3.2. Qualitative Results

– Thematic analysis for the free-response question: “What does animal welfare mean to you?”

Initial review of survey responses identified six themes: Relationship and Role of Humans, Needs of the Animal, Promoting Positive States, Preventing Negative States, Five Freedoms/Domains, and Role of the Animal. All theme definitions and examples of each theme are shown in Table 4. After initial coding meetings, the Five Freedoms/Domains theme was integrated into the Needs of an Animal theme primarily due to its rare occurrence. The coding was complex as approximately 25% of the responses had three or more themes and only 25% of the responses were coded as one single theme. A very small percentage (~5%) of responses included all five themes.

Relationship and Role of Humans. This theme was the most commonly occurring theme across all survey responses appearing in over 80% of responses. Although the majority of the responses were coded with multiple themes, the most common single theme was the Relationship and Role of Humans, representing approximately 25% of the total responses.

Needs of the Animal. This theme included any need of the animal; this included basic needs such as shelter, food and water, and emotional needs such as socialization and mental stimulation. Needs of an Animal were mentioned in 30% of the total responses and was almost always found with other themes within a response; this theme only occurred in 6 responses as the only theme present. Mentions of needs were both broad and specific.

Promoting Positive States. All instances of this theme included mention of a positive state or condition of the animal. This theme occurred in approximately half of the responses.

Preventing Negative States. There was only one survey response that was singly coded with this theme; all other appearances of this theme occurred in tandem with multiple other themes. Additionally, this was the least commonly found theme, appearing in under 20% of the responses. Phrases coded with this theme were related to reducing negative experiences of the animals, and often this was related to the Relationship and Role of Humans theme.

Role of the Animal. This theme was almost always found with at least one other theme; there were only two instances of this theme being the sole theme for a survey response. This theme was found in approximately 20% of all responses. Phrases coded as this theme often referred to the use of the animal and/or the specific environment of the animal.

– Thematic analysis for three free-response questions: “In your opinion, what does an (1) agricultural animal, (2) dog or cat, and (3) horse or other equid need in order to have a good life?”

Initial review of survey responses identified nine themes. After initial coding meetings, one theme was collapsed into two other themes and final coding used seven themes: Basic Needs, Social and Emotional Needs, Health Needs, Environment, End of Life, Human Responsibility and Interaction, and Five Freedoms/Domains. All theme definitions and examples of each theme are shown in Table 5. The coding was complex as approximately 26% to 30% of the responses had three or more themes and less than 10% of the responses for each animal category were coded as one single theme. A very small percentage (~1%) of responses for each animal type included six themes.

Basic Needs. This theme was the most commonly occurring theme across each animal category, included in over 80% of responses. This theme was defined by the authors as mentions of food, water, or shelter. Responses coded with this theme also included references to diet and nutrition or specific types of food such as forage. Ninety percent (556) of responses within the agricultural animal category question included the mention of basic needs highlighting how respondents perceived the importance of basic needs as a foundational component of animal welfare.

Social and Emotional Needs. This category included many different parameters related to the social and emotional needs of animals. The frequency of mention varied slightly across animal categories but was present in close to two-thirds of all responses for all categories (62.3%, 385 agricultural animal; 68.1%, 425 dog or cat; 66.9%, 410 horse or other equid). Across all animal categories, there was frequent mention of providing “mental stimulation,” and some examples include: “to offer mental stimulation to prevent boredom and stereotypic behaviors” and “some type of fun and stimulation.” There were several comments suggesting that companion animals needed a different level of mental stimulation as compared with agricultural animals, specifically (e.g., “they also need more mental stimulation and interaction than agricultural animals”). Specific types of stimulation were also mentioned including things like socialization, activity (varying by animal category), and enrichment. Although there were responses related to enrichment for all animal categories, the dog or cat question responses often had specific details regarding the types of behaviors that were important to promote with enrichment: “proper enrichment, opportunities for catch/chase/hunt behavior,” and “partake in activities that allows them to exhibit their natural behaviors, like chasing a mouse toy or using a snuffle mat.” Additionally, for dogs and cats when enrichment was mentioned, there was often inclusion of stating that “toys” and “play time” were beneficial. The opportunity to socialize and/or have companions and groups was often noted: “opportunity for social interaction with conspecifics,” “to have buddies,” “the ability to interact with others of the same species,” and “socialization (if social species).” There was also some mention of social interactions specific to the category of animal. For example, the concept of a herd was found in agricultural animal responses: “they need the sense of having a herd just like it would in the wild.” The need for exercise, a job/purpose, or doing work was mentioned frequently for horses and other equids but not present for other animal categories: “some type of stimulation like riding or place to run/do work,” “a normal amount of stimulation and riding,” “the addition of a job or more intensive way to move/exercise than livestock,” and “they also require a purpose.”

Health Needs. This theme included general statements about maintaining health and providing veterinary care in addition to specific mentions of health requirements. This theme was present in less than half of the responses for all animal categories: 42.9%, 265 for agricultural animals; 37.3%, 233 for dogs and cats; and 38.7%, 237 for horses and other equids. One difference that emerged across animal categories was the inclusion of hoof and teeth care in horses that was not present in the two other categories: “proper care of hooves and teeth,” “teeth floating,” “proper farrier and vet appointments,” and “vaccinations, worming and pest prevention plan, regular vet, dentist and farrier care.”

Environment. This category included comments related to the environments of the animals including both descriptions of the environment itself in addition to what the environment needed to provide for the animal. Simple shelter was not included in this category as it was classified as a Basic Need. This theme was found in over half of the responses for dogs and cats and horses and other equids (51.9%, 324 and 60.5%, 371, respectively). This theme was mentioned in two-thirds (67.0%, 414) of the responses for agricultural animals. For all animal categories there was frequent mention of needing “space.” Depending on the category of animal, this space may have been described differently. Very commonly in the agricultural and horse questions, space was mentioned in reference to having the “space to”: “run,” “roam,” “walk around,” “move,” and “graze.” The space was sometimes described as “adequate,” “enough,” or “plentiful.” In dogs and cats, space was focused on “places to relax/hide,” “their own space,” “comfortable place to sleep,” and “adequate space for at least part of their day to do as much activity as they want to do.” The agricultural animal responses had multiple mentions of the environment being safe: “guaranteed safety from predators and the elements,” “a safe location that is free of predators and hazards,” and “safe spaces from predators.” Lastly, an environmental aspect that was unique within the dog and cat responses was the mention of a “loving home.” The concept of home was not mentioned in the other two animal categories.

End of Life. This theme included mention of the welfare of an animal at the end of its life with the most frequent mentions being euthanasia and slaughter. This theme was mentioned rarely in the dog and cat (0.6%, 4) and horse or other equid (0.8%, 5) categories. Although still infrequent as compared to other themes, it was most commonly found in the agricultural animal question (6.5%, 40). Most of the responses coded as this theme for agricultural animals were related to slaughter.

Human Responsibility and Interaction. This theme included both the mention of the role of the human to ensure welfare of the animal and the relationship between the animal and the human. This theme was present most often in the dog and cat question (60.1%, 375), followed by the horse or other equid (39.3%, 241) and agricultural animal questions (18.9%, 117). All animal categories included some responses that were simply about providing care to the animals, such as: “gentle handling,” “proper care,” and “humane treatment,” and this was generally what was found in the agricultural animal question. All animal categories also had some responses simply stating that “human interaction” was a need. The dog and cat responses included more examples of animals needing love, attention, and companionship from an owner: “compassion from their owners,” “provide them with love,” “companionship with their owner,” “love and attention”, and “a loving family.” For the horse and other equine question, there was considerable mention of riding and/or training as an important part of human care and responsibility, which was not present in the other animal categories: “training for handling and pleasure riding,” “some kind of behavioral training,” “personalized work/training plan tailored to the horse,” and “a good trainer.”

Five Freedoms/Domains. Some of the responses included specific mention of the Five Freedoms or Five Domains. This type of response did not occur frequently but was identified more frequently in agricultural animals (3.1%, 19) as compared with dogs and cats and horses or other equids (1.4%, 9 and 1.8%, 11, respectively). Some of the responses coded with this theme listed out the Five Freedoms and others just made a reference to the framework itself.

Reference to Prior Answer. Due to the similarity of the questions, the percentage of responses that referenced a previous answer increased across the three animal categories. These responses simply referenced prior answers (e.g., “basically the same as an agricultural animal,” “about the same as cats and dogs”, and sometimes “same as above” or “same as previous 2”). Prior answers were referenced in approximately 20% of the response for dogs and cats (18.8%, 117) and horses or other equids (20.7%, 127). The agricultural animal question was first so there was no reference to a prior answer in that animal category.

## 4. Discussion

The objective of the current study was to evaluate perceptions and attitudes about animal welfare of students enrolled in animal science programs at institutions across the United States and determine if welfare considerations differ across animal categories (e.g., agricultural animals, cats and dogs, horses and other equids, research animals, and wildlife). While a total of 624 individuals completed the online survey, the authors are unable to calculate a specific response rate due to the nature of survey distribution. Respondents represented 24 institutions, indicating not all universities that received the study chose to distribute it. The probable reasons for this are numerous (e.g., avoiding sending too many emails, not receiving the email, highly supportive of the topic, not supportive of the topic) and could have introduced some bias into the study population. As seen in the results, the general sentiment of the survey population towards animal welfare topics was positive; future work should consider ways to engage individuals more fully in these discussions, particularly regarding those that may have different opinions or be less willing to share perspectives through a survey format. The potential selection bias of this sample population is important to consider when reviewing the results of the study; individuals who chose to take the survey could be more interested in and potentially more informed about animal welfare which could impact their responses.

It is important to call attention to the fact that the majority of respondents indicated that animal welfare was important across all animal categories. This finding is reflective of societal values regarding how animals should be treated. These results are in line with the notion that animal welfare is a public good, such that improved animal welfare or reduction of animal suffering is something all members of society can benefit from [35]. The responsibility for improving animal welfare is shared by all members of society as improved welfare benefits all, regardless of their interaction with and use of animals [3,36,37,38]. This notion supports the broad appreciation for animal welfare across animal categories found in this study. Although most participants agreed welfare was important for conducting research with animals and wildlife, the proportion of agreement within this category was slightly less which may be due to differences in respondent experience across animal categories. The relatively greater proportion of respondents that indicated that welfare is an important consideration for agricultural animal production, owning a cat or dog, owning a horse or other equid, and while using animals for research compared to those that agreed with the statement for wildlife may be, in part, due to the proximity of the respondents to these specific animal categories; to illustrate, 70% of U.S. households own a pet, as reported in a recent nationwide pet owners survey [39], and greater than 95% of Americans consume animal-derived proteins [40]. It is possible that due to the connection of the public with these animal categories (to varying capacities), individuals have a greater sense of awareness and knowledge of the needs of these animals and feel a greater sense of responsibility for their welfare. In contrast, exposure to wildlife is limited for most individuals, which may begin to explain why there is less agreement on the importance of this animal category with livestock, pets, horses, and research animals in this survey. The survey did not define “wildlife,” and therefore that could have led to different interpretations about what type of animals were included (e.g., exotic animals in zoos compared to wild animals in national parks). Moreover, perceptions regarding the importance of animal welfare for wildlife could be due to limited human-wildlife interactions that people have, resulting in a decreased sense of responsibility for this animal category. Alternatively, the decrease in agreement could be due to the tools currently used to assess animal welfare which stress human interventions for welfare improvement [41,42]. Since human interventions to improve wildlife welfare are more ambiguous using the current assessment tools, it is possible students do not initially consider this animal category when thinking about welfare.

One unique aspect of this survey is the integration of both quantitative and qualitative responses to construct a comprehensive illustration of animal welfare perceptions of the study population. The survey incorporated various welfare aspects found in many of the foundational welfare frameworks, and free-response questions provided insight into what survey respondents felt were important components of animal welfare for different animal categories.

One of the themes identified from free-response questions focusing on needs for various animal categories was the Five Freedoms/Domains. Authors anticipated having a greater frequency of this theme, as Five Freedoms is a foundational construct used to conceptualize animal welfare needs that has existed for decades [43]. Interestingly, direct mention of these frameworks (which included an exact naming of the framework or quotation of all freedoms/domains) was relatively low across animal categories. There were components of the Five Freedoms noted throughout many of the responses but generally only one or two “freedoms” were included in the response and those responses were coded accordingly (i.e., as either a Basic need or an Emotional and Social need). In quantitative analysis of survey questions, the majority of respondents identified the listed freedoms (e.g., freedom from fear and distress, freedom from injury and disease) as important to animal welfare across animal categories. The Five Freedoms [43] are used across animal industries in policy statements and programmatic documents for animal welfare and are particularly abundant within the food production industry (e.g., Nestle [44]; American Humane [45]; ASPCA [46]; Cargill [47]; GRSB [48]); thus, it is not surprising that many individuals are familiar with this concept.

As animal welfare science advances, there have been new animal welfare constructs that have been developed, one of those being the Five Domains. The Five Domains model was developed almost 30 years ago [49], although it has been frequently updated [50,51,52,53,54] and more recently, integrated into animal welfare programming documents for companies and associations [55,56]. Although the Five Domains framework is growing in popularity and familiarity, this framework is still relatively new, particularly in the livestock welfare space. One prominent component of the Five Domains is the focus on promoting positive welfare states [53]. Traditionally, reducing negative experiences has been a focus of welfare improvement; this approach can be seen in the majority of the Five Freedoms. As animal welfare science progresses and evolves there has been a considerable shift in trying to ensure animals also have positive experiences. Another feature of the Five Domains is the emphasis that the physical and functional domains (e.g., malnutrition, exposure, and disease) directly impact affective elements (e.g., hunger, thirst, and fear) that impact overall welfare [57]. Although the Five Domains were not specifically mentioned frequently in the qualitative analysis, social and emotional needs were noted in approximately two-thirds of responses across animal types with many of those responses specifically mentioning the importance of mental stimulation, again, highlighting the essence of the Five Domains being identified even if not directly stated. Additionally, an observation that was persistent across responses in the qualitative analysis was a trend in discussing the addition of positive stimuli rather than the prevention or removal of negative stimuli to improve welfare.

Although animal welfare is a critical consideration in all animal industries, it is of heightened focus and continues to grow in agricultural animal production [58,59]. The results from this study highlight some of the differences in perception of welfare across animal categories. For each of the twelve factors related to animal welfare that were provided in the survey, which was not an exhaustive list but one that included many different aspects of welfare needs, the majority of respondents considered the factors as extremely important (75.1%) or very important (18.4%) for dogs or cats and horses or other equids. For eleven of the twelve factors, there was a decrease in the percentage of respondents who rated the factors as extremely or very important for food and fiber animals. These results demonstrate that although animal welfare is perceived as important, most factors in this study were viewed as markedly less important for food and fiber animals. Past research indicates that many different factors can impact perceptions of welfare for this animal category [60]. For example, two studies of veterinary students found those who plan to work with food and fiber animals tended to have reduced empathy towards this animal category and were more willing to perform potentially painful procedures on these animals [60,61]. Additionally, experience in a farm or rural context and being male have also been shown to decrease perceptions of welfare importance for this animal category [32,60,62], identifying that there are numerous factors that may influence individual perceptions about animal welfare.

The decrease in the percentage of respondents ranking the twelve welfare factors as extremely or very important was consistent across all factors except one: freedom from thirst. Additionally, a common theme across both the qualitative and quantitative results was the importance of basic needs regardless of the animal category. Basic needs as a theme in the qualitative analysis was most often described as food, water, and shelter, i.e., the basics needed for survival. In human psychology, Maslow’s hierarchy of needs [63] is a well-known motivational theory displayed as a pyramid whose base is established by physiological needs, i.e., as basic needs are met (e.g., physiological and safety needs), the higher needs in the pyramid emerge (e.g., love, belonging, and self-actualization). Provision of basic needs is paramount to creating a foundation of animal welfare regardless of animal category. The importance of food and water can also be seen in the Five Freedoms as the first freedom addresses the need to eliminate hunger and thirst. In production animal agriculture, meeting nutritional, health, and environmental needs is integral to animal care [64], and thus, perhaps the nuance in the differences in perceptions about welfare across animal categories is in the higher needs. Although basic needs seem to be the basis of animal welfare perception (and survival), the topic is clearly more nuanced as 75% of survey respondents in this study identified two or more welfare themes in their free-responses. The combination of multiple themes in the qualitative responses supports that students’ current perceptions of welfare are multifaceted and complex, extending beyond only basic needs.

In response to the open-ended question, “What does animal welfare mean to you?” over 80% of respondents emphasized human–animal interactions (HAI) as an essential component of welfare. Participants highlighted not only humane handling of animals but also discussed humans’ relationships with their animals. The respondents’ ability to identify the role of humans as an element necessary for positive animal welfare aligns with a recent shift in the literature underscoring the important impact human behavior can have on animal well-being. Human factors such as age, gender, occupational stress, social support, knowledge and experience, empathy for people and animals, well-being, ability to recognize pain, attitudes towards animals, locus of control, and personality have all been found to influence animal welfare via HAIs [54,57,65,66,67,68,69]. Although these factors have an indirect influence (e.g., increased human empathy can lead to gentler animal handling which decreases animal stress) on welfare, Mellor [57] argues that humans have almost complete control over many direct aspects of animal welfare such as access to water and food, the space in which the animals reside, and the environmental complexity and social groupings for domestic and captive wildlife animals. While HAIs have been identified as a central component of animal welfare, respondents in this study reported that the need for human interaction differed based on animal category.

Although the majority of participants identified HAIs as an essential element of animal welfare, when asked to identify specific animal needs, clear differences between animal categories emerged. Approximately 60% of participants mentioned human interaction as an essential need for companion animals. However, human interaction was only identified as a specific need 40% of the time for equids and only 20% of the time for agricultural animals. The language the respondents used to describe the type of human interaction varied between animal categories as well. When discussing companion animals, participants highlighted this category’s need for human love and attention, identifying these animals as valued members of the family. The type of language used and the frequency with which human interaction was mentioned for this animal category is likely due to participants’ experiences of the benefits derived from interacting with animals in this category. Companion animals have been shown to benefit human health via increased physical activity, increased social connectedness, and reduced feelings of loneliness and isolation [70,71,72,73]. Given the reliance of humans on companion animals to fulfill both physical and emotional needs, it is understandable that participants consistently mentioned human interaction for this category and did so with strong emotional language. Equids were also perceived as needing human interaction however, the language used to describe necessary human interactions with this animal category was markedly less emotional and focused on the qualifications of the human necessary to properly care for animals in this category. The human–equid relationship is thought to be complex as horses are often perceived as something between livestock and companion animals [64]. When discussing the needs of agricultural animals, human interaction was mentioned infrequently compared to the other animal categories and the responses focused on reducing negative interactions with humans with a few participants highlighting the need for regular human contact. Although the development of needs and standards of animal care (e.g., Animal Welfare Act of 1966 [74]; the Five Freedoms [43]) were created to prevent the poor treatment of animals, research indicates that animals in this category benefit not only from the reduction of negative HAIs but also through repeated, positive human interactions [68,69,75]. Regular, positive interactions have been shown to reduce stress, increase the number of offspring, improve disease resistance, and increase productivity in agricultural animals [67,68]. Future research should seek to understand why many students do not perceive human interaction as an essential need for agricultural animals.

In qualitative analysis of questions highlighting the needs for different animal categories, a theme that was present, although infrequent, was end-of-life. This theme was identified when respondents mentioned the welfare of an animal near the end of its life, including references to slaughter (for agricultural animals) and euthanasia (for dogs or cats and horses or other equids). Considering that producers and consumers of animal-derived proteins increasingly value the welfare of animals in the food production chain [76], the low frequency of this theme in the qualitative analysis, particularly for the agricultural animal category, was somewhat unexpected. Even though the World Organization for Animal Health (OIE) incorporates the condition in which an animal both lives and dies into its definition of animal welfare [77], other widely accepted definitions of animal welfare emphasize an animal’s welfare during its lifetime rather than at its death. Further, the relationship between death and welfare is complex, since the notion of ending an animal’s life seems counterintuitive to maintaining its welfare state. The authors postulate that respondents may not perceive death as part of their welfare definitions, thus contributing to the limited number of responses mentioning this theme for any animal category. Interestingly, the most frequent mention of this theme, while still limited, pertained to agricultural animals. The greater proportion of respondents who mentioned the end-of-life theme for agricultural animals compared to any other category may be the result of respondents associating food animals with death more commonly than for companion animals; slaughter is a critical endpoint for animals in the food production chain [76] and could have been at the forefront of respondents’ minds when answering this question about food animal species. Additionally, the infrequent occurrence of this theme across all animal categories may be due to the fact that end-of-life considerations are typically sensitive and challenging matters [78,79,80], further underlined by the lack of responses discussing the importance of euthanasia or slaughter in the present study.

In the suite of questions asking about the importance of various welfare factors, the two factors that received the most “Not at all” or “Slightly important” responses were “having a life worth living” and the “ability for choice and control within their environment.” Both statements are from two different animal welfare concepts that have not been as widely referenced or adopted in the livestock industries in the United States. The concept of a “life worth living” once again focuses on the importance of positive experiences in relation to an animal’s quality of life; to have a life worth living an animal must have more positive experiences than negative [81]. This concept was developed by the Farm Animal Welfare Council in a report meant to evaluate progress in farm animal welfare in Great Britain since the Five Freedoms were established [43]. The relatively greater frequency of these responses could simply be reflective of the lack of understanding of what this concept of a “life worth living” actually means, as other answers within the survey reflect support for this concept of a good quality of life. The other answer that was less identified as important, “the ability for choice and control,” is a component of a separate framework which is another adaptation of the Five Freedoms called the Five Opportunities to Thrive [82]. This concept was developed as a mechanism to optimize animal welfare management for captive animals by assessing opportunities (i.e., inputs into the system) for animals and their subsequent welfare impacts (i.e., outcome-based measures; [82,83]). Again, this concept has not permeated the livestock industry, but it is seen more frequently within the exotic animal and conservation space [84]. Four of the “opportunities” are highly similar to the Five Freedoms (e.g., opportunity for a well-balanced diet, opportunity for optimal health) but the “opportunity for choice and control” is more unique, focusing on the importance of having the option to exert some control by making choices in a captive environment, such as decisions about space use or social interactions [83], which has been shown to reduce stress [84,85,86,87] and thus, improve animal welfare. Authors speculate that these two concepts will become more integrated into the animal welfare lexicon as the focus on improving animal welfare continues to grow and evolve.

In addition to looking at differences across animal categories, authors also sought to explore perceptions about the welfare status of different species within the agricultural animal category. The majority of respondents agreed that for dairy cattle, beef cattle, and sheep and goats (68.1%; 67.5%; 62.8%, respectively) “predominant methods used to raise [them] provide an appropriate level of animal welfare.” These findings align with several survey studies that have found overall perceptions of food and/or fiber animal welfare are generally positive, particularly regarding standard production practices for beef and dairy animals (veterinary faculty, [88]; animal science faculty, [15]; consumers, [4]). Nevertheless, perceptions of animal welfare in the present study varied across animal species, which was reflected in fewer respondents agreeing that the ways in which swine (52.1%) and poultry (43.0%) are currently raised provide appropriate welfare. These data lend support to other work that has identified that individuals perceive intensively managed animals (i.e., layers, broilers, and swine) as having poorer welfare than those raised in extensively managed systems (i.e., beef and sheep) [4,15]. The authors ascertain that as the intensity in which animals are reared increases, whether that be through housing, environment, management, or production efficiency, perceptions of animal welfare and acceptance of standard production practices tend to decrease. This relationship can be noted in many of the animal advocacy initiatives that have emerged over the past several years; many of the undercover videos created by activist groups that have emerged about poor welfare practices and conditions have focused on intensely raised pigs and poultry, potentially contributing to the greater percentage of students who perceived swine and poultry species to have decreased animal welfare compared to beef and dairy cattle. Additionally, social media has been cited as a resource in several survey studies that is used to gather information related to animal welfare [21,28]; thus, it is important to consider the influence of social media and other forums on public knowledge and perceptions of animal welfare.

## 5. Conclusions

While animal welfare is an important topic that has widespread impacts, including influencing animal usage laws and impacting consumers’ purchasing decisions, public perceptions of animal welfare are complex and understudied. Perceptions of animal welfare have been shown to vary between species and between production system types, congruent with the findings in the current study. Understanding animal science students’ perceptions of animal welfare is particularly important, both because many animal science programs lack formal animal welfare education components and because animal science students will potentially work in the agricultural and animal sectors, managing animal welfare issues as part of their careers. In general, the results of this survey suggest most respondents agree that animal welfare is important for all species, but clear perception differences emerged between animal categories when asked about specific welfare needs. The provision of basic needs and human interaction was recognized as a necessity of welfare across animal categories, although the frequency of and nature of mention was less frequent in agricultural animals. Future research should explore how these differences in perception of welfare across animal categories impact welfare assessments and the adoption of new management practices and interventions.

## Figures and Tables

**Table 1:** The percentage of respondents that selected “strongly agree” or “agree” in response to the question “animal welfare is an important consideration” for each of the following: agricultural animal production, owning a cat or dog, owning a horse or other equid, conducting research with animals, and for wildlife.

	Specific Animal Category or Use (%, *n*/*n*)				
	Agricultural Animal Production	Owning a Cat or Dog	Owning a Horse or Other Equid	Conducting Research with Animals	Wildlife
Animal welfare is an important consideration for:	99% ^a^, 618/624	99% ^a^, 619/624	99% ^a^, 621/623	99% ^a^, 617/623	97% ^b^, 608/624

Note: Categories with different superscripts (a,b) differ (*p* < 0.05).

**Table 2:** The percentage of respondents for each level of importance when asked “what is your assessment of the importance of each of these parameters for” three different animal categories (i.e., food and fiber animals, dogs and cats, and equids) for 12 factors related to animal welfare. A chi-square test was performed for each species within a factor related to welfare; one *p*-value for each factor is shown for clarity. χ2 between 224 and 1566 (df between 2 and 4; *n* between 608 and 624; *p* < 0.0001; Table 2); effect sizes ranged from 0.6 to 1.6, indicating a large effect size.

Factor Related to Welfare	Extremely Important	Very Important	Moderately Important	Slightly Important	Not at All	*p*-Value for Chi-Square Tests within a Factor
*Room to move around*						
Food and fiber (*n* = 624)	55%, 345	27%, 171	15%, 92	3%, 16	0	<0.0001
Dog or cat (*n* = 624)	72%, 446	24%, 149	5%, 28	0.2%, 1	0	
Equids (*n* = 619)	82%, 509	15%, 91	3%, 18	0.2%, 1	0	
*Freedom to express normal behaviors*						
Food and fiber (*n* = 624)	62%, 388	29%, 180	8%, 52	0.5%, 3	0.2%, 1	<0.0001
Dog or cat (*n* = 622)	71%, 442	23%, 141	6%, 39	0	0	
Equids (*n* = 619)	75%, 463	22%, 135	3%, 21	0	0	
*Having a sufficient and comfortable area to lie down*						
Food and fiber (*n* = 624)	71%, 444	26%, 160	3%, 17	0.5%, 3	0	<0.0001
Dog or cat (*n* = 623)	79%, 494	19%, 120	1%, 9	0	0	
Equids (*n* = 619)	75%, 467	22%, 134	3%, 18	0	0	
*Freedom from fear and distress*						
Food and fiber (*n* = 621)	71%, 443	23%, 144	5%, 32	0.3%, 2	0	<0.0001
Dog or cat (*n* = 623)	83%, 516	15%, 92	2%, 15	0	0	
Equids (*n* = 618)	81%, 502	16%, 96	3%, 19	0.2%, 1	0	
*Having positive interactions with humans*						
Food and fiber (*n* = 622)	54%, 337	27%, 169	15%, 94	3%, 21	0.2%, 1	<0.0001
Dog or cat (*n* = 621)	84%, 520	14%, 89	2%, 12	0	0	
Equids (*n* = 618)	76%, 468	19%, 118	4%, 27	0.8%, 5	0	
*Freedom from injury and disease*						
Food and fiber (*n* = 622)	82%, 512	16%, 100	2%, 10	0	0	<0.0001
Dog or cat (*n* = 622)	86%, 535	13%, 81	1%, 6	0	0	
Equids (*n* = 620)	85%, 528	13%, 79	2%, 13	0	0	
*Freedom from hunger*						
Food and fiber (*n* = 624)	88%, 550	11%, 68	1%, 6	0	0	<0.0001
Dog or cat (*n* = 622)	90%, 560	9%, 55	1%, 7	0	0	
Equids (*n* = 620)	90%, 557	9%, 56	1%, 6	0.2%, 1	0	
*Freedom from thirst*						
Food and fiber (*n* = 624)	91.2%, 569	8%, 51	0.6%, 4	0	0	<0.0001
Dog or cat (*n* = 623)	91.8%, 572	8%, 49	0.3%, 2	0	0	
Equids (*n* = 620)	91%, 564	8%, 52	0.7%, 4	0	0	
*A painless death*						
Food and fiber (*n* = 621)	78%, 483	19%, 117	3%, 16	0.8%, 5	0	<0.0001
Dog or cat (*n* = 621)	83%, 514	14%, 84	3%, 18	0.6%, 4	0.2%, 1	
Equids (*n* = 620)	82%, 508	15%, 91	3%, 17	0.5%, 3	0.2%, 1	
*Having a life worth living*						
Food and fiber (*n* = 611)	52%. 320	28%, 172	15%, 89	4%, 22	1%, 8	<0.0001
Dog or cat (*n* = 612)	74%, 452	20%, 121	5%, 28	2%, 9	0.3%, 2	
Equids (*n* = 608)	66%, 403	26%, 155	6%, 39	2%, 9	0.3%, 2	
*Ability for choice and control within their environment*						
Food and fiber (*n* = 616)	34%, 207	27%, 166	28%, 172	9%, 58	2%, 13	<0.0001
Dog or cat (*n* = 621)	49%, 301	29%, 181	17%, 105	4%, 26	1%, 8	
Equids (*n* = 611)	49%, 297	29%, 176	17%, 104	5%, 28	1%, 6	

Note: The italics differentiates the 12 factors.

**Table 3:** The percentage of respondents for each level of agreement in response to the statement: “Predominant methods presently used to raise each animal type below for food and/or fiber provide an appropriate level of animal welfare.” Respondents who selected “strongly agree” or “agree” were combined and represented as “Agreed;” respondents who selected “neither agree or disagree,” “somewhat disagree,” or “strongly disagree” were combined and represented as “Did not agree” (*n* = 621).

Response Category	Animal Categories				
	Dairy Cattle	Beef Cattle	Poultry	Swine	Sheep and Goats
Agreed	68.1% ^a^, 425	67.5% ^a^, 421	43.0% ^b^, 268	52.1% ^c^, 325	62.8% ^d^, 392
Did not agree	26.1% ^a^, 163	26.3% ^a^, 164	49.7% ^b^, 310	39.7% ^c^, 248	19.4% ^d^, 121
Did not have enough information to decide	5.8% ^a^, 36	6.3% ^a^, 39	7.4% ^b^, 46	8.2% ^c^, 51	17.8% ^d^, 111

Note: Different superscripts (a,b,c,d) within a row indicate *p* < 0.01.

**Table 4:** Theme definitions, main concepts within each theme, and a sample of quotations from responses to the free-response survey question: “what does animal welfare mean to you?” (*n* = 624).

Themes and Definitions	Main Concepts	Primary Examples	Frequency ^1^ %, *n*
Relationship and Role of Humans: Any reference to the relationship between animals and humans (e.g., caretakers and owners), including what humans are expected to provide for the animals.	Having a good relationship; treating them right; treating animals with respect; people’s responsibility towards animals; stockmanship, stewardship, and husbandry; providing protection and safety; ethical obligation; fair or humane treatment	*“Animal welfare involves the relationship people have with animals and how people should make sure they are treated as humanely and compassionately and possible.”* *“Animal welfare means…treating animals with reverence and gratitude rather than solely seeking a result or end product from them.”* *“Ensuring the animals are treated with respect as living beings…”* *“Good animal welfare encompasses good husbandry techniques…”* *“The care and protection of animals.”* *“Animal welfare is the humane and ethical treatment of animals.”*	81.4%, 508
Needs of the Animal: A physical, environmental, social or emotional need of an animal	Basic needs including food, water, and shelter; socialization with other animals; allowing animals to express natural behaviors; medical needs; direct mention of the Five Freedoms was included in this category (mentioned infrequently)	*“To me, animal welfare means providing a safe, comfortable environment to animals. This includes adequate food, water, shelter, clean air, space, etc..”* *“Animal welfare means that animals…have at least the basic necessities (food, water, shelter) provided.”* *“Meeting all behavioral needs.”* *“Providing the animal with the five freedoms during its life.”* *“…to provide them with enrichment where possible.”*	30.8%, 192
Promoting Positive States: Adding general benefit to an animal’s life and promoting a positive state	Good quality of life; good well-being; comfort; safety; good health	*“…means providing them a quality of life worth living.* *“It means animals having the best life they can.”* *“Animal welfare is the humane treatment of animals that promotes their health in the best possible way.”* *“Animal welfare to me is consideration of the total wellbeing of the animal.”* *“The safety and wellbeing of animals.”*	53.8%, 336
Preventing Negative States: Removing detriments to the animal’s life	Reducing and minimizing the occurrence of states such as pain and suffering, neglect, disease, injury, and stress	*“Animal welfare is the overall care of animals so as not to inflict unnecessary pain or suffering.”* *“…meaning they aren’t being abused or neglected.”* *“…prevention of disease, free of fear (to the best of the caretaker’s ability).”*	18.1%, 113
Role of the Animal: Statement about the type of animal or its job.	Mentioning the role or animal type such as companion animal, animal used in research, or an animal used for food	*“Animals are treated humanely while being used to fulfill a purpose to society, such as food and/or fiber.”* *“The protection of animals’ mental and physical wellbeing as it pertains to their role in our industry whether that is working animals in the field, companions, or production animals.”* *“Animal welfare is the well-being and treatment of animals whether it be as livestock, as a pet, or as an experimental animal.”*	20.5%, 128

Note: The italics show direct quotes.^1^ The number of times the code was found across all survey responses was counted and divided by the total number of survey responses (*n* = 624).

**Table 5:** Theme definitions, main concepts within each theme, and a sample of quotations for each theme from responses to the questions: In your opinion, what does an ((1) agricultural animal (cattle, sheep, goat, pig, or poultry intended for food and fiber use), (2) dog or cat, and (3) horse or other equid) need in order to have a good life? In the “Animal Category” column, the letter A denotes agricultural animal, C denotes dog or cat, and E denotes horse or other equid.

Themes and Definitions	Main Concepts	Animal Category ^1^	Primary Examples	Frequency ^1^ %, *n*
Basic needs: Mentions of food, water, and shelter	Indicating one or more of the following is required: food, water, and shelter. Stating basic needs/necessities are required, sometimes including “at a minimum.”	A	*“a diet that contains all necessary nutrients,* *water”* *“need good food and always have access to* *water”* *“basic needs such as shelter, food, water”*	90.0%, 556
		C	*“a healthy diet with treats, plenty of water”* *“have access to adequate food, water and* *shelter”* *“adequate/appropriate amounts of food, water”*	83.0%, 518
		E	*“nutrient rich food, clean water, shelter”* *“will need a diet to fit their needs”* *“they need access to water at all times and should be fed on a regular basis”*	80.9%, 496
Social and Emotional needs: Mentions of emotional needs, social group, companionship, and mental stimulation	This was a broad category and included things like: social group, companionship, mental stimulation, enrichment, performing natural behaviors, promoting positive emotions and reducing negative states (i.e., suffering, stress), getting exercise, having a routine, having choice, and having a job or purpose.	A	*“social interactions”* *“enrichment items to enhance their ability to express natural behaviors”* *“to avoid negative states such as frustration, boredom, or fear”*	62.3%, 385
		C	*“enrichment activities to keep their minds stimulated”* *“stimulation such as toys or exercise”* *“companionship”*	68.1%, 425
		E	*“adequate exercise, mental stimulation”* *“display normal roaming behaviors”* *the opportunity to work and rest, depending on what it has been trained to do”*	66.9%, 410
Health needs: Any mention of veterinary care, medical needs/care, or maintaining a health state	This included general statements about maintaining health or providing veterinary care; specific examples of health requirements were also included such as: vaccinations, dewormers, medication; statements about preventing sickness and injury	A	*“veterinary care”* *“medical care when necessary”* *“available precautions against sickness and disease (dewormers/ vaccines/ preventatives)”*	42.9%, 265
		C	*“checked regularly by a veterinarian”* *“proper medical care and proactive treatment such as vaccines”* *“healthcare”*	37.3%, 233
		E	*“proper attention to hooves and teeth, protection from physical harm and illness”* *“taking the proper precautionary health measures such as vaccines, supplements, deworming, and antibiotics, based on breed and use”* *“medical needs, good farrier work”*	38.7%, 237
Environment: Any description of elements of an animal’s environment	This included descriptions of the environment, outside of simply stating shelter; reference to housing, open space, room to do certain things; living conditions; pasture; a home	A	*“good housing that is correct for the living conditions ie warm housing in the winter; open housing in the summer”* *“they need plenty of space”* *“access to open spaces”* *“clean and dry bedding, a space of their own, cleanliness”*	67.0%, 414
		C	*“space to exercise”* *“a warm dry place to sleep”* *“proper space and housing”*	51.9%, 324
		E	*“large enough paddock”* *“a place to run”* *“somewhere comfortable to sleep”*	60.5%, 371
End-of-life: Any description of the end of an animal’s life	Humane death; euthanasia; slaughter	A	*“timely euthanasia or stunning before slaughter that painlessly and instantly renders the animal insensible”* *“humanitarian slaughter—free of pain and stress”* *“humane slaughter when needed”*	6.5%, 40
		C	*“freedom from pain and suffering even in death”* *“low stress euthanasia, if needed”* *“humane euthanasia when needed”*	0.6%, 4
		E	*“low stress euthanasia, if needed”* *“humane end-of-life”* *“humane euthanasia when needed”*	0.8%, 5
Human responsibility and interaction: Mention of the role of the human or the relationship between the animal and the human	Proper and humane care, handling, and treatment from humans/owners/producers; the importance of the relationship with the animal and the owner; providing love, attention, and compassion to the animal; educated owners who have the ability and means to care for the animal	A	*“gentle handling”* *“should not be abused, well taken care of”* *“regular interaction with humans”*	18.9%, 117
		C	*“lots of attention”* *“loving family to care for them”* *“grooming”* *“should be treated as part of the family”*	60.1%, 375
		E	*“compassion and a good trainer”* *“need an owner that will keep them well-groomed”* *“human interaction”*	39.3%, 241
Five Freedoms/Domains:	Specific mention of the Five Domains or Five Freedoms either by providing the title or listing all of the domains/freedoms	A	*“Basically the five freedoms proposed: Freedom from hunger and thirst, freedom from discomfort, from pain, injuries and diseases, freedom to express their normal behavior, freedom from fear and distress.”*	3.1%, 19
		C	*“deserves all the 5 freedoms”* *“Five freedoms+”* *“their 5 freedoms”*	1.4%, 9
		E	*“they need the five freedoms”*	1.8%, 11

Note: The italics show direct quotes.^1^ The number of times the code was found across all survey responses was counted and divided by the total number of survey responses.

## Data Availability

Data available upon request to the corresponding author.

## Supplementary Materials

The following supporting information can be downloaded at: https://www.mdpi.com/article/10.3390/ani12172294/s1, Survey S1.
