# Supplementary material for: A Nationwide Survey of Animal Science Students’ Perceptions of Animal Welfare across Different Animal Categories at Institutions in the United States
Source: Animals (Basel). 2022 Sep 5;12(17):2294. doi: 10.3390/ani12172294 (PMC9454941; doi:10.3390/ani12172294)
Supplement: Supplementary file 1 [file animals-12-02294-s001.zip › animals-1874850-supplementary Survey S1..pdf]

The consent form is not included in this document.

Questions

What does animal welfare mean to you?

In your opinion, what does an agricultural animal (cattle, sheep, goat, pig, or poultry intended for food and fiber use) need in order to have a good life?

In your opinion, what does a dog or cat need in order to have a good life?

In your opinion, what does a horse or other equid need in order to have a good life?

What are the top 3 animal welfare challenges that you think are relevant to agricultural animal production?

Challenge #1

Challenge #2

Challenge #3

What are the top 3 animal welfare challenges that you think are relevant to companion animals (cats, dogs, hamsters, etc)?

Challenge #1

Challenge #2

Challenge #3

I feel confident I know how to research and discuss an animal welfare topic, even one that I know very little about, in order to form an educated opinion that I can communicate to others.

- ○ Strongly agree
- ○ Somewhat agree
- ○ Neither agree nor disagree
- ○ Somewhat disagree
- ○ Strongly disagree

If you had a question about animal welfare, which of the following resources would you use? Select all that apply.

- ☐ Peer-reviewed publications
- ☐ Extension website
- ☐ Livestock/veterinary associations
- ☐ Humane Society of the United States
- ☐ General web search
- ☐ Social media
- ☐ Peers or relatives
- ☐ Other, please specify ________________

At your university, which of the following types of education or opportunities are offered for animal welfare? Choose all that apply.

	Within your Animal Science Department	Outside your Animal Science Department	I don't know
Animal welfare judging team	○	○	○
Animal welfare club	○	○	○
Animal welfare course	○	○	○
Animal behavior course	○	○	○
Combination animal	○	○	○

welfare/animal	behavior course		
Graduate degree program in Animal Welfare	○	○	○

Have you taken an animal welfare course?

- ○ Yes
- ○ No

Was it at your current university?

- ○ Yes
- ○ No

It is important to have an animal welfare course as part of the animal science curriculum.

- ○ Strongly agree
- ○ Somewhat agree
- ○ Neither agree nor disagree
- ○ Somewhat disagree
- ○ Strongly disagree

Animal welfare is an important component of my education.

- ○ Strongly agree
- ○ Somewhat agree
- ○ Neither agree nor disagree
- ○ Somewhat disagree
- ○ Strongly disagree

Animal welfare courses would be helpful for my future career.

- Strongly agree
- Somewhat agree
- Neither agree nor disagree
- Somewhat disagree
- Strongly disagree

Concepts related to animal welfare are integrated in other courses in my degree.

- Strongly agree
- Somewhat agree
- Neither agree nor disagree
- Somewhat disagree
- Strongly disagree

Rate your confidence level in discussing the following components of animal welfare.

	Very confident	Fairly confident	Somewhat confident	Slightly confident	Not at all confident
Health	○	○	○	○	○
Productivity	○	○	○	○	○
Behavior	○	○	○	○	○
Pain	○	○	○	○	○
	Very confident	Fairly confident	Somewhat confident	Slightly confident	Not at all confident
Emotional states	○	○	○	○	○
Ethics/societalconcerns	○	○	○	○	○

If I was faced with an animal welfare dilemma, I would feed confident addressing the issues.

- Strongly agree
- Somewhat agree
- Neither agree nor disagree
- Somewhat disagree
- Strongly disagree

Animal welfare is an important consideration in agricultural animal production.

- Strongly agree
- Somewhat agree
- Neither agree nor disagree
- Somewhat disagree
- Strongly disagree

Animal welfare is an important consideration for owning a cat or dog.

- Strongly agree
- Somewhat agree
- Neither agree nor disagree
- Somewhat disagree
- Strongly disagree

Animal welfare is an important consideration for owning a horse or other equid.

- Strongly agree
- Somewhat agree
- Neither agree nor disagree
- Somewhat disagree
- Strongly disagree

Animal welfare is an important consideration when conducting research with animals.

- Strongly agree
- Somewhat agree
- Neither agree nor disagree
- Somewhat disagree
- Strongly disagree

Animal welfare is an important consideration for wildlife.

- Strongly agree
- Somewhat agree
- Neither agree nor disagree
- Somewhat disagree
- Strongly disagree

If you were to take an animal welfare course, which of the following attributes would be most important? Select all that apply.

- Ethical discussions
- Hands-on application
- Practical, applied questions
- Understanding species differences
- Discussing current hot topics in animal welfare
- Other, please specify

What is your assessment of the importance of each of these parameters for animals raised for food and fiber?

	Extremely important	Very important	Moderately important	Slightly important	Not at all important	I don't know

Room to move aroundfreely

Freedom to expressnormal behaviors

Having a sufficient and comfortable area to lie down

Freedom from fearand distress

Having positive interactions with humans

Freedom from injuryand disease

Freedom from hunger

Freedom from thirst

Freedom from painand discomfort

A painless death

Having a life worthliving

Ability for choice and control in their environment

What is your assessment of the importance of each of these parameters for a dog or cat?

	Extremely important	Very important	Moderately important	Slightly important	Not at all important	I don't know

Room to move aroundfreely

Freedom to expressnormal behaviors

Having a sufficient and comfortable area to lie down

Freedom from fearand distress

	Extremely important	Very important	Moderately important	Slightly important	Not at all important	I don't know

Having positive interactions with humans

Freedom from injuryand disease

Freedom from hunger

Freedom from thirst

Freedom from painand discomfort

A painless death

Having a life worthlibing

Ability for choice and control in their environment

What is your assessment of the importance of each of these parameters for horses or other equids?

	Extremely important	Very important	Moderately important	Slightly important	Not at all important	I don't know

Room to move aroundfreely

Freedom to expressnormal behaviors

Having a sufficient and comfortable area to lie down

Freedom from fearand distress

Having positive interactions with humans

Freedom from injuryand disease

Freedom from hunger

Freedom from thirst

Freedom from painand discomfort

A painless death

	Extremely important	Very important	Moderately important	Slightly important	Not at all important	I don't know
Having a life worthliving						
Ability for choice and control in their environment						

Select your level of agreement with this statement. Predominant methods presently used to raise each animal type below for food and/or fiber provide an appropriate level of animal welfare.

	Strongly	Somewhat	Neither agree nor	Somewhat	Strongly	I don't have enough information

	agree	agree	disagree	disagree	disagree	to decide
Dairy cattle	○	○	○	○	○	○
Beef cattle	○	○	○	○	○	○
Poultry	○	○	○	○	○	○
Swine	○	○	○	○	○	○
Sheep and goats	○	○	○	○	○	○

Is there anything else about your perspectives on animal welfare that you would like to share?

Demographics

How old are you?

- ○ 18-24
- ○ 25-34
- ○ 35-44
- ○ 45-54
- ○ 55-64
- ○ 65 and older
- ○ Decline to answer

What gender do you identify with?

- ○ Man
- ○ Woman
- ○ Non-binary
- ○ Other
- ○ Decline to answer

How would you best describe yourself?

American Indian or Alaska Native

- ○
- ○ Asian
- ○ Black or African American
- ○ Native Hawaiian or Other Pacific Islander
- ○ White
- ○ [ ] Other, please specify:
- ○ Decline to answer

Do you identify as Hispanic, Latino/a or Spanish?

- ○ Yes
- ○ No
- ○ [ ] Other, please specify:
- ○ Decline to answer

In what state or US territory have you lived the longest?

[ ]

What type of community have you lived in for most of your life?

- ○ Suburban
- ○ Rural
- ○ Urban
- ○ [ ] Other, please specify:
- ○ Not defined

How would you best describe your dietary preference?

- ○ Non-vegetarian
- ○ Vegetarian
- ○ Vegan
- ○ [ ] Other, please specify:
- ○ Not defined

Which university do you currently attend?

What is your current year in university?

- Undergraduate (years 1-5)
- Masters
- PhD
- Post Doc
- Other, please specify:

What is the general area of your major? Select all apply

- Animal/Livestock Science
- Dairy Science
- Poultry Science
- Equine Science
- Other, please specify:

What is your area of specialization?

What are your career plans after graduation?

Which animals do you have experience either raising or working with in the past? Select all that apply.

- Cats or dogs
- Small companion animal (e.g., mouse, hamster, rat)
- Exotic animal (e.g., chinchilla, sugar glider)
- Reptile or amphibian
- Fish
- Livestock (e.g., cows, sheep, pigs, etc.)
- Horses and other equids

- ☐
- ☐ Poultry (e.g., chickens, turkeys, etc.)
- ☐ Laboratory animals in a research setting
- ☐ Wildlife
- ☐ ________ Other, please specify:

Which animals would you like to get experience working with in the future? Select all that apply.

- ☐ Companion animals (e.g. cats and dogs)
- ☐ Livestock (e.g., cows, sheep, pigs, etc.)
- ☐ Horses and other equids
- ☐ Poultry (e.g., chickens, turkeys, etc)
- ☐ Laboratory animals in a research setting
- ☐ Wildlife
- ☐ ________ Other, please specify:
